# P-642. A High Prevalence of Liver Steatosis and Metabolic Syndrome Among Hospitalized Patients with Community-acquired Pneumonia

**DOI:** 10.1093/ofid/ofae631.839

**Published:** 2025-01-29

**Authors:** Branimir Gjurasin, Juraj Krznaric, Nina Vrsaljko, Neven Papic

**Affiliations:** University Hospital for Infectious Diseases Zagreb, Zagreb, Grad Zagreb, Croatia; University hospital for infectious diseases Zagreb, Zagreb, Grad Zagreb, Croatia; University hospital for infectious diseases Zagreb, Zagreb, Grad Zagreb, Croatia; University Hospital for Infectious Diseases Zagreb, Zagreb, Grad Zagreb, Croatia

## Abstract

**Background:**

Community-acquired pneumonia (CAP) is one of the leading causes of morbidity and mortality. While metabolic syndrome has been identified as a risk factor for CAP outcomes, the role of metabolic dysfunction-associated steatotic liver disease (MASLD) in CAP is less clear. This study aimed to investigate the prevalence and severity of MASLD in hospitalized patients with CAP.

Prevalence of metabolic syndrome and MASLD in patients with community-acquired pneumonia

**Figure:**
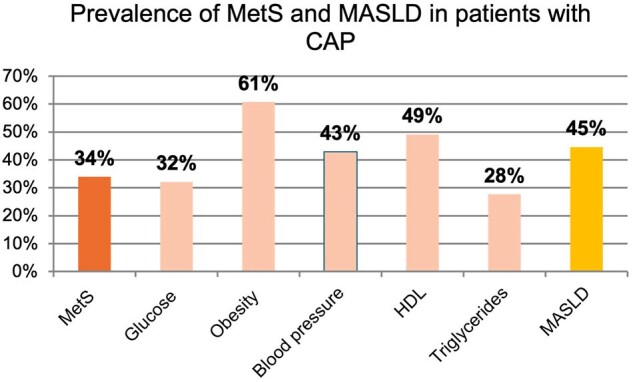

**Methods:**

A prospective observational cohort study (SepsisFAT) included hospitalized adult patients with CAP at three clinical departments at the University Hospital for Infectious Diseases Zagreb. At admission, patients were screened for metabolic syndrome, the degree of liver steatosis was estimated using the controlled attenuation parameter and patients were subesquently diagnosed with MASLD according to current guidelines.

**Results:**

One hundred and fifty patients were included (86 males, median age of 63, IQR 51-73 years). The prevalence of MASLD was 45% (n=68) and metabolic syndrome of 31% (n=46). 43 (29%) patients had insulin resistance, 74 (49%) arterial hypertension, HDL < 1.03 or < 1.29 mmol/L 77 (51%) patients, 46 (31%) triglycerides ≥ 1.7 mmol/L and central adiposity 80 (53%). Steatosis was graded as mild in 21 (14%), moderate in 16 (11%) and severe in 31 (21%) patients, and 20 (13%) patients had fibroleastography > 9.0kPa. The prevalence of MASLD was higher in the severe than non-severe CAP group (43% vs. 34%).

**Conclusion:**

The prevalence of MASLD among hospitalized patients with CAP is higher than estimated in the general population, with the highest prevalence in severe CAP treated at the intensive care unit. This highlights the need for further studies to examine the clinical impact of MASLD on CAP outcomes.

**Disclosures:**

**All Authors**: No reported disclosures

